# Environmental risk factors for autism: an evidence-based review of systematic reviews and meta-analyses

**DOI:** 10.1186/s13229-017-0121-4

**Published:** 2017-03-17

**Authors:** Amirhossein Modabbernia, Eva Velthorst, Abraham Reichenberg

**Affiliations:** 1 Department of Psychiatry and Seaver Autism Center, Icahn School of Medicine at Mount Sinai, New York, USA; 2 Department of Preventive Medicine, Icahn School of Medicine at Mount Sinai, New York, USA; 3 Friedman Brain Institute, Department of Psychiatry, Icahn School of Medicine at Mount Sinai, New York, USA; 4 Seaver Autism Center, Department of Psychiatry, Icahn School of Medicine at Mount Sinai, New York, USA

**Keywords:** Autism spectrum disorders, Environment, Epigenetics, Gene-environment interaction, Metals, Nutrition, Pregnancy prenatal, Toxin, Vaccine

## Abstract

**Background:**

According to recent evidence, up to 40–50% of variance in autism spectrum disorder (ASD) liability might be determined by environmental factors. In the present paper, we conducted a review of systematic reviews and meta-analyses of environmental risk factors for ASD. We assessed each review for quality of evidence and provided a brief overview of putative mechanisms of environmental risk factors for ASD.

**Findings:**

Current evidence suggests that several environmental factors including vaccination, maternal smoking, thimerosal exposure, and most likely assisted reproductive technologies are unrelated to risk of ASD. On the contrary, advanced parental age is associated with higher risk of ASD. Birth complications that are associated with trauma or ischemia and hypoxia have also shown strong links to ASD, whereas other pregnancy-related factors such as maternal obesity, maternal diabetes, and caesarian section have shown a less strong (but significant) association with risk of ASD. The reviews on nutritional elements have been inconclusive about the detrimental effects of deficiency in folic acid and omega 3, but vitamin D seems to be deficient in patients with ASD. The studies on toxic elements have been largely limited by their design, but there is enough evidence for the association between some heavy metals (most important inorganic mercury and lead) and ASD that warrants further investigation. Mechanisms of the association between environmental factors and ASD are debated but might include non-causative association (including confounding), gene-related effect, oxidative stress, inflammation, hypoxia/ischemia, endocrine disruption, neurotransmitter alterations, and interference with signaling pathways.

**Conclusions:**

Compared to genetic studies of ASD, studies of environmental risk factors are in their infancy and have significant methodological limitations. Future studies of ASD risk factors would benefit from a developmental psychopathology approach, prospective design, precise exposure measurement, reliable timing of exposure in relation to critical developmental periods and should take into account the dynamic interplay between gene and environment by using genetically informed designs.

**Electronic supplementary material:**

The online version of this article (doi:10.1186/s13229-017-0121-4) contains supplementary material, which is available to authorized users.

## Background

Autism spectrum disorder (ASD) is a group of neurodevelopmental disorders characterized by persistent impairment in social communication and interaction and restricted and repetitive patterns of behavior, interests, or activities [1]. There is evidence that one in every 132 to one in every 68 individual suffers from ASD [2, 3]. According to a recent meta-analysis, ASD accounted for 7.7 million disability adjusted life years in 2010 and was the leading mental cause of disability in children under five in terms of years lived with disability [2].

While in the majority of cases of the exact etiology of ASD remains unknown, novel technologies and large population-based studies have provided new insight into the risk architecture of ASD and the possible role of environmental factors in etiology [4]. Twin studies provide a unique platform to study the relative contribution of genetic and (shared and non-shared) environmental factors to the variability of a certain trait or disorder. Dizygotic (DZ) twins on average share 50% of their genes, and monozygotic (MZ) twins share 100% of their genes. Twins are matched for many characteristics including age, in utero and family environment, and various aspects of early and late development. Taken together, these features allow for estimating heritability (phenotypic variation that is attributable to the genotypic variation) of ASD by taking into the account its covariance within MZ and DZ twins [5]. Earlier twin studies suggested heritability as high as 80–90% for ASD with little contribution from the environment [4, 6, 7]. However, according to recent evidence, up to 40–50% of variance in ASD liability is determined by environmental factors [8–13]. Newer studies of monozygotic twins have yielded concordance rates of <50%, with lower concordance for dizygotic twins, suggesting that both genes and environment play roles in the development of ASD [4, 14–16]. Given the vulnerability of the developing brain to environmental factors, the causative association between environmental factors and ASD is biologically plausible [17]. Furthermore, historical proof-of-concept evidence shows a causal relation between specific environmental risk factors such as thalidomide and misoprostol and ASD [17–20].

In the present paper, we provide an evidence-based review of the current knowledge about environmental risk factors in ASD using findings from published systematic reviews and meta-analyses. Although reviewing systematic reviews and meta-analyses inherently focuses on the most well-studied findings, it automatically excludes the studies that are published after the included reviews or that, for any reason, are not systematically reviewed. It is therefore important to keep in mind that the purpose of this review is not to review all possible environmental risk factors, but rather to provide a wide view of the evidence landscape in epidemiology of risk factors for ASD.

## Methods

We searched Pubmed since inception until December 2016 for ((autis*) or (“pervasive developmental disorder”) or (“Asperger”)) and (“systematic review” or “meta-analysis”). We extracted the following for meta-analyses: number of studies, study design, imprecision (>0.25 difference between effect estimates and their upper or lower confidence bound for dichotomous variables and >0.5 difference for continuous outcomes), inconsistency (presence of heterogeneity *I* ^2^ > 50% or *P* value of heterogeneity test < 0.10), magnitude of association (relative risk estimate <2.0 small, 2.0–5.0 medium, >5.0 large), publication bias, and indirectness (that is measuring exposure based on population-level assessment) [21, 22]. Imprecision reflects how wide or narrow the confidence interval is: the narrower the confidence interval, the greater the precision. Heterogeneity or inconsistency refers to the degree of between-study variability in a meta-analysis.

A meta-analysis provides higher quality of evidence for an association if it yielded precise, consistent, direct, and strong association without publication bias. For systematic reviews, we narratively summarized the authors’ conclusion in a separate table (because quantitative assessment was not possible). For each potential risk factor, the latest systematic review or meta-analysis was considered unless indicated otherwise (See Additional file 1: Table S1 for more details).

## Results

A total of 663 citations were found. One additional relevant reference was found through manual search of the reference list in the remaining papers. Of these, 584 records were excluded by title or abstract. Of the remaining 80 studies, 32 references (9 qualitative systematic reviews and 23 meta-analyses) were included for the purpose of the review (See Additional file 1: Figure S1 and Table S1 for the details on the excluded studies).

**Fig. 1:**
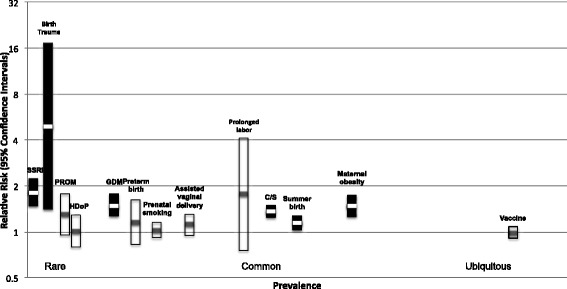
The association between several environmental factors and autism spectrum disorders; C/S, caesarian section; GDM, gestational diabetes mellitus; HDoP, hypertensive disease of pregnancy; PROM, premature rupture of membrane; SSRI, selective serotonin reuptake inhibitor

Figure 1 and Tables 1 and 2 summarize the findings of meta-analyses and systematic reviews respectively. Below, we present the evidence for nine different groups of risk factors (including about 100 individual risk factors), namely advanced parental age, pregnancy-related factors, prenatal medication, maternal diseases, nutrition, environmental toxins, vaccine, maternal smoking, and maternal immigration.

**Table 1:** Summary of meta-analyses of environmental risk factors for autism spectrum disorders

Risk factor [ref], year	No. of studies	Study design	Estimates	Precision	Consistency	Directness	Publication bias
Advanced parental age [23], 2017							
• Highest paternal age category	20	4 cohort; 16 case-control	+	+	−	+	Absent
• Highest maternal age category	19	4 cohort; 15 case-control	+	+	−	+	Absent
Labor [25], 2011							
• Prolonged labor	9	NA	+/−	−	−	+	Absent
• Induced or augmented labor	8	NA	+/−	−	−	+	Absent
• Precipitous labor	5	NA	+/−	−	−	+	Absent
• Premature rupture of membranes	7	NA	+/−	−	+	+	Absent
Delivery options [25], 2011							
• Cesarean section [29], 2015	21	6 Cohort; 15 case-control	+	+	−	+	Absent
• Emergency cesarean	4	NA	+/−	−	−	+	Absent
• Elective cesarean	2	NA	+/−	−	−	+	Absent
• Delivery anesthesia	7	NA	+/−	−	+	+	Absent
• General anesthesia	3	NA	+/−	−	+	+	Absent
• Assisted vaginal delivery	14	NA	+/−	+	+	+	Absent
• Forceps	7	NA	+/−	−	+	+	Absent
• Vacuum extraction	2	NA	−/+	−	−	+	Absent
Conditions at birth [25], 2011							
• Abnormal presentation	15	NA	+	−	−	+	Absent
• Breech presentation	4	NA	+	−	+	+	Absent
• Cord complications	14	NA	+	−	+	+	Absent
• Fetal distress	4	NA	+	−	+	+	Absent
• Birth injury or trauma	6	NA	++	−	+	+	Absent
• Twins or multiple birth	10	NA	+	−	−	+	Absent
Maternal hemorrhage [25], 2011	4	NA	++	−	+	+	Absent
Timing of birth [25], 2011							
• January through March	4	NA	+/−	+	+	+	Absent
• April through June	4	NA	+/−	−	−	+	Absent
• July through September	4	NA	+/−	+	+	+	Absent
• October through December	4	NA	−/+	−	−	+	Absent^b^
• Fall	3	NA	−/+	−	−	+	Absent
• Winter	3	NA	−/+	−	−	+	Absent
• Spring	3	NA	−/+	−	−	+	Absent
• Summer	3	NA	+	+	+	+	Absent
Birth spacing (ref ≥36 m) [30], 2016	7	3 cohort; 3 cross-sectional; 1 case-control					
• <12 months	5	NA	+	−	−	+	NC
• 12–23 months	5	NA	+/−	−	−	+	NC
• 24–35 months	5	NA	−/+	−	−	+	NC
Birth spacing (ref 24–59 m) [30], 2016							
• <12 months	4	NA	+	−	−	−	NC
• 12–23 months	4	NA	+	−	−	−	NC
• >60 months	4	NA	+	−	−	−	NC
Gestational age [25], 2011							
• Postterm	14	NA	+/−	−	−	+	Absent
• Preterm	17	NA	+/−	−	−	+	Absent
• >4 weeks preterm	2	NA	+/−	−	+	+	Absent
Birth weight [25], 2011							
• Birth weight <2500 g	15	NA	+	−	−	+	Absent
• Birth weight <2000 g	2	NA	+/−	−	−	+	Absent
• Birth weight <1500 g	3	NA	++	−	+	+	Absent
• Birth weight >4000 g	6	NA	+/−	−	+	+	Absent^b^
Neonatal clinical and medical conditions [25], 2011							
• Meconium aspirated	3	NA	+++	−	+	+	Absent^b^
• Neonatal infection	2	NA	−/+	−	+	+	Absent
• Elevated temperature	2	NA	+/−	−	−	+	Absent
• ABO or Rh incompatible	5	NA	++	−	+	+	Absent
• Hyperbilirubinemia or jaundice	14	NA	+/−	−	−	+	Absent
• Jaundice	4	NA	+/−	−	+	+	Absent
• Hyperbilirubinemia	6	NA	+	−	−	+	Absent
• Phototherapy	2	NA	+/−	−	−	+	Absent
• Medical intervention in the first month	7	NA	+/−	−	−	+	Absent
Impaired gas exchange [28], 2016							
• Acidosis at birth	2	2 population-based	+	+	+	+	Absent
• Apgar at 1 min <6	3	3 population-based	++	−	+	+	Absent
• Apgar at 1 min <7	3	3 population-based	+	+	+	+	Absent
• Apgar at 5 min <7	6	6 population-based	+	−	+	+	Absent
• Apgar at 5 min <8	3	2 population-based; 1 clinic-based	++	−	+	+	Absent
• Apgar at 5 min <9	2	1 population-based; 1 clinic-based	+/−	−	+	+	Absent
• Apnea/delayed crying	5	3 population-based; 2 clinic-based	++	−	−	+	Absent
• Respiratory distress	12	6 population-based; 6 clinic-based	+	+	+	+	Absent
• Ventilation/O2 treatment	9	4 population-based; 5 clinic-based	++	−	+	+	Present^c^
• Undefined hypoxia/asphyxia	9	2 population-based; 7 clinic-based	++	−	−	+	Absent
Mother born in another country [24], 2009	5	NA	+/−	−	−	+	Absent
Previous fetal loss [24], 2009	13	NA	+/−	−	+	+	Absent
Birth order/parity [24], 2009							
• 1 pregnancy increase	8	NA	−/+	+	−	+	Absent
• 1st vs. not 1st	11	NA	+/−	+	−	+	Absent
• 1st vs. 2nd	4	NA	+/−	−	−	+	Absent
• 1st vs. 2nd or 3rd	6	NA	+/−	−	−	+	Absent
• 1st vs. 3rd+	4	NA	+	+	+	+	Absent
• 1st vs. 4th+	6	NA	−/+	−	−	+	Absent
• 1st or 4th vs. 2nd or 3rd	5	NA	+/−	−	−	+	Absent
• 4th vs. 2nd or 3rd	5	NA	+/−	−	+	+	Absent
Maternal illness/conditions [24], 2009							
• Maternal proteinuria	3	NA	−/+	−	+	+	Absent
• Toxemia/preeclampsia, hypertension, swelling	25	NA	+/−	−	−	+	Absent
• Maternal diabetes [31], 2014	12	3 cohort	+	+	+	+	Absent
		9 case-control	+	−	+	+	Absent
• Maternal infection [33], 2016	15	2 cohort; 13 case-control	+	+	−	+	Absent
• Bacterial infection [33], 2016	4	NA	+	+	−	+	Absent
• Viral infection [33], 2016	4	NA	+/−	−	−	+	Absent
• Influenza [33], 2016	3	NA	+/	+	−	+	Absent
• Genitourinary infection [33], 2016	8	NA	+	+	+	+	Absent
• Skin infection [33], 2016	3	NA	+	−	+	+	Absent
• Gastrointestinal infection [33], 2016	3	NA	+/−	−	+	+	Absent
• Respiratory infection [33], 2016	7	NA	+/−	+	+	+	Absent
• Family history of autoimmune disease [35], 2015	11	3 cohort; 6 case-control; 2 cross-sectional	+	+	−	+	Present^c^
• Maternal autoimmune disease [34], 2016	10	9 case-control; 1 cohort	+	+	+	+	Absent
• Rubella	3	NA	+/−	−	+	+	Absent
• Fever	4	NA	+/−	−	+	+	Absent
• Nausea vomiting	6	NA	+/−	−	−	+	Absent
• Physical injury accident	5	NA	+/−	−	+	+	Absent
• Any illness	4	NA	+/−	−	+	+	Absent
Maternal weight [32], 2016							
• Maternal underweight	5	All cohort	+/−	+	+	+	Absent
• Maternal overweight	5	All cohort	+	+	+	+	Absent
• Maternal obesity	7	6 cohort; 1 case-control	+	−	−	+	Absent
Prenatal visit [24], 2009	2	NA	−/+	−	−	+	Absent
Bleeding [24], 2009	19	NA	+	−	−	+	Absent
• 1st trimester	2	NA	+/−	−	+	+	Absent
• 2nd trimester	2	NA	−/+	−	+	+	Absent
• 3rd trimester	2	NA	−/+	−	+	+	Absent
Placental abnormalities [24], 2009	8	NA	+/−	−	+	+	Absent
• Placenta previa	2	NA	+/−	−	+	+	Absent
• Placenta abruption	2	NA	−/+	−	+	+	Absent
• Placental infarcts	2	NA	+/−	−	+	+	Absent
High maternal weight gain during pregnancy [24], 2009	5	NA	−/+	−	−	+	Absent
Maternal smoking [39], 2015	15	4 cohort; 11 case-control	+/−	+	−	+	Absent
• Prenatal	8	NA	+/−	+	−	+	NC
• Neonatal	2	NA	−	+	+	+	NC
• Postnatal	5	NA	+	−	−	+	NC
Medication use during pregnancy [24], 2009	15	NA	+	−	+	+	
• Anticonvulsants	2	NA	+/−	−	+	+	Absent
• SSRIs [38], 2016	8	3 cohort; 5 case-control	+	−	+	+	Absent
• Antidepressants [112], 2014	3	All case-control	+/−	−	−	+	Absent
Thimerosal [47], 2014	9	1 retrospective and 2 prospective cohort; 6 case-control	+/−	+	+	Mixed^a^	Present^d^
Inorganic mercury [47], 2014	3	3 case-control	+	−	+	+	NC
Vaccination [52], 2014	10	5 cohort	−/+	+	+	+	Absent
		5 case-control	−	+	−	+	Absent
MMR vaccine [52], 2014	3	All cohort	−/+	+	+	+	Absent
Metal exposure [48], 2014							
• Hg through vaccine [52], 2014	2	Both retrospective cohort	+/−				
• Hair Hg	7	All case-control	+/−	−	NC	+	NC
• Hair Cd	4	All case-control	−/+	−	NC	+	NC
• Hair Pb	5	All case-control	+	−	NC	+	NC
• Hair Cu	4	All case-control	+/−	−	NC	+	NC
• Hair Se	3	All case-control	−/+	−	NC	+	NC
• Hair Li	3	All case-control	−/+	−	NC	+	NC
• Zn/Cu [113], 2015	11	All case-control	−/+	−	−	+	Absent
Nutritional intake [40], 2013							
• Zinc [41], 2016	12	All case-control	−	+	−	+	Absent
• Calcium	8	NA	−−	−	−	−	Absent
• Carbohydrates	7	NA	−/+	−	−	−	Absent
• Energy	6	NA	−/+	−	−	−	Absent
• Fiber	6	NA	+/−	−	−	−	Absent
• Iron	7	NA	+/−	−	−	−	Absent
• Protein	7	NA	−−	−	−	−	Absent
• Total fat	6	NA	+/−	−	−	−	Absent
• Vitamin A	6	NA	−/+	−	−	−	Absent
• Vitamin C	7	NA	−/+	−	−	−	Absent
• Vitamin D [42], 2016	11	All case-control	−−−	−	−	+	Possible
• Vitamin E	5	NA	+/−	−	−	−	Absent
Air pollution [46], 2016							
• PM10 (every 10 μg/m^3^)	6	5 case-control; 1 cohort	+	+	−	−	NC
• PM2.5 (every 10 μg/m^3^)	3	3 case-control	+	+	−	−	NC
• O3 (every 10 μg/m^3^) [114], 2014	2	2 case-control	+	+	+	−	NC

+/−, positive but statistically non-significant effect estimates; +/, marginally positive; −/+, negative but statistically non-significant effect estimates; +, positive statistically significant effect estimates (number of + corresponds to the strengths of the association); −, negative statistically significant effect estimates (number of − corresponds to the strengths of the association)

^a^Only direct −/+

^b^Begg’s test was not significant; Egger’s test was significant

^c^Adjusted for bias +

^d^Adjusted for bias −/+

*NA* not available, *NC* not calculated

**Table 2:** Summary of systematic reviews of environmental risk factors for autism spectrum disorders

Risk factors	Design	Summary of findings	Comments
Maternal immigration [53], 2015	10 population-based	The adjusted results showed higher rates of ASD if children had migrant mothers (2.69%) compared with children of non-migrant mothers (0.91%). Six out of ten studies found that giving birth postmaternal migration significantly raised risks of ASD, with the exception of children born to Hispanic migrant mothers who had lower rates of ASD (compared to all other migrant groups)	A meta-analysis by another group supports these findings
Preconceptional chemical exposure [49], 2014	3 case-control	All studies showed an increased rate of ASD in children whose parents were exposed to chemicals	The studies were limited by retrospective design and small sample sizes
Pesticide exposure during pregnancy [49], 2014	1 retrospective case-control; 3 prospective cohort	All showed an increased rate of ASD in children with gestational exposure to chemicals including two prospective studies of organophosphates	The studies provide enough evidence to justify larger studies of pesticide exposure in ASD
Pesticide exposure during childhood [49], 2014	1 retrospective cross-sectional; 1 prospective cohort; 1 computer model	One study showed an association between organophosphate exposure; one between DDE exposure and ASD, and one between phosphine exposure and ASD	
Air pollution during prenatal period [49], 2014	6 retrospective	All studies showed an increased rate of ASD associated with air pollution (with particulate matter in three studies and with NO2 in two studies)	A meta-analysis of four prospective European cohorts showed no association between air pollution and ASD
Air pollution during childhood [49], 2014	3 retrospective case-control	All studies showed some association between air pollution and ASD (association with NO2 is replicated in two studies)	
Exposure to other chemicals [49], 2014	2 prospective cohort; 4 retrospective case-control	No effect in prospective studies; retrospective studies found some effects for solvents and other toxicants	Retrospective studies used questionnaires
Toxic waste sites [49], 2014	2 case series, ecological	Association of Superfund site with ASD in both studies	
Water pollutant [49], 2014	2 ecological	No association between water chlorination and ASD in either studies	
Heavy metals [49], 2014	8 ecological	7 studies showed association between Hg and ASD, one showed no association	Ecological studies are limited by indirect measures of exposure
In-house flooring [49], 2014	1 cohort	Polyvinyl chloride vs. wood floor increases the risk of ASD	
Mercury levels [49], 2014	29 case-control	12 studies with elevation in at least one tissue in patients with ASD	Studies measuring levels of heavy metals were limited by cross-sectional design and small sample size. Furthermore, it is unclear how much cross-sectional measurements of metals in the body reflect the causal effect of such exposures on ASD risk.
Lead levels [49], 2014	25 case-control	11 studies with elevation in at least one tissue in patients with ASD	
Cadmium levels [49], 2014	14 case-control	5 studies with elevation in at least one tissue in patients with ASD	
Aluminum levels [49], 2014	11 case-control	3 studies with elevation in at least one tissue in patients with ASD	
Arsenic [49], 2014	8 case-control	5 studies with elevation in at least one tissue in patients with ASD	
Nickel [49], 2014	3 case-control	2 studies with elevation in at least one tissue in patients with ASD	
Uranium levels [49], 2014	2 case-control	1 studies with elevation in at least one tissue in patients with ASD	
Tin levels [49], 2014	1 case-control	1 studies with elevation in at least one tissue in patients with ASD	
Heavy metals and severity [49], 2014	7 correlational studies	All studies showed some correlation between ASD severity and heavy metal levels (particularly Hg and Pb)	These studies suggest a dose-response relationship between metal exposure and ASD.
Heavy metals and chelation [49], 2014	12 uncontrolled studies	All showed improvement in symptoms of ASD following chelation treatment	The studies are limited by lack of the control group
Urine porphyrin studies [49], 2014	4 case-series; 7 case-control	All studies showed some association with severity, prediction, or oxidative stress	The studies are limited by retrospective nature
Solvents, pesticides, and PCB [49], 2014	2 case series; 2 case-control	Case series showed levels above normal adult range for children with ASD; case-control studies showed no association between PCB and ASD	The studies were limited by either lack of proper control group or small sample size
Phthalate [51], 2016	2 cohort; 3 case-control	Three studies showed association between phthalate exposure and ASD; two studies showed no association	
PBDE [49], 2014	1 case-control	PBDE resulted in higher activation of immune response in patients with ASD	
Valproate [37], 2014	1 population-based prospective; 2 prospective case series; 1 retrospective	All studies found significant and strong association between prenatal valproate exposure and ASD	The association between valproate and ASD is likely to be causal given the strength of association and evidence for biological plausibility
Folic acid [44], 2016	11 studies	The findings were inconsistent; some studies provide support for the beneficial effect of folic acid on risk of ASD, whereas others show the opposite effect	Observational studies were limited by self-report, whereas RCTs were limited by use of multivitamin
Camel milk [115], 2016	2 small short-term RCTs	Results showed beneficial effects on ASD symptoms	It is unclear whether this benefit is clinical and whether it extends to longer period of treatment
Casein and gluten-free diet [116], 2014	16 studies	Findings are inconsistent	Studies with positive findings were mostly of low quality
Omega-3 [43], 2011	2 RCTs	Studies found no evidence for beneficial effects of omega-3 on ASD symptoms	
Assisted reproductive technologies [36], 2013	2 cohort; 5 case-control	3 studies showed positive association between ART and ASD, 3 studies showed no association, and 1 study showed a negative association between ART and ASD. The authors concluded that at present, no evidence supports the association between ART and ASD	There are several sources of heterogeneity such as study design, exposure definition, data source, and confounders.

*ART* assisted reproductive technology, *ASD* autism spectrum disorder, *DDE* dichlorodiphenyldichloroethylene, *NO2* nitrogen dioxide, *RCT* randomized controlled trial

### Advanced parental age

Wu et al. [23] performed a meta-analysis of 27 studies on association between advanced parental age and ASD. They showed that every 10-year increase in maternal and paternal age increases the risk of ASD in the offspring by 18 and 21% respectively. Furthermore, compared with the reference age group, oldest age category (in both mothers and fathers) was associated with a small but significant and precise increase in risk of ASD in the offspring.

### Pregnancy-related complications and conditions

Several systematic reviews and meta-analyses have summarized the evidence on the association between pregnancy complications and ASD [24–27] (Tables 1 and 2). Gardener et al. [24, 25] carried out two comprehensive reviews of prenatal and perinatal risk factors for ASD. They found statistically significant pooled estimates with small effect size for abnormal presentation, breech presentation, cord complications, fetal distress, multiple births, low birth weight, small for gestational age, congenital malformations, hyperbilirubinemia, and earlier birth (first vs. third born). Evidence suggested a medium yet imprecise effect size for five risk factors including maternal hemorrhage, Rh or ABO incompatibility, birth injury or trauma, birth weight <1500 g, and feeding difficulties at birth. Large and imprecise effect sizes were observed for neonatal anemia and meconium aspiration (Table 1).

A meta-analysis by our group has specifically addressed the link between proxies of impaired gas exchange and ASD [28]. Out of ten proxies that were analyzed, the effect size was medium (and imprecise) for apnea/delayed crying, undefined hypoxia/asphyxia, 1-min Apgar score <6, 5-min Apgar score <8, and O2 treatment, and small and imprecise for the rest (Table 1).

Curran et al. [29] reviewed 21 studies of the association between cesarean section (C/S) and ASD. They found a 36% increase in the risk of ASD following C/S that was reduced after adjusting for confounders or limiting the analysis to population-based studies and was absent in the cohort studies. The effect size was small, precise, and inconsistent. Moreover, they showed that estimates were lower for elective C/S compared with emergency C/S (Table 1).

In a systematic review of seven studies, Conde-Agudelo and colleagues found that birth spacing <12 months and >60 months are associated with a significantly increased risk of ASD (Table 2) compared to the birth spacing in the middle range. The estimates were of small magnitude, inconsistent, and imprecise. However, the included studies were generally of high quality. The estimates were slightly larger for the former subtype autistic disorder [30] (Table 1).

Xu and colleagues conducted a meta-analysis on the association between maternal diabetes and ASD in the offspring. For cohort studies, they found a 74 and 43% (both small effect sizes) increase in risk for pregestational and gestational diabetes respectively (Table 1) [31]. As expected, slightly higher odds ratios were observed for case-control studies. In a meta-analysis of seven studies, Wang and colleagues [32] showed that overweight and obese mothers (but not underweight mothers) have an increased risk of ASD by about 28 and 36% respectively. The increased risk for overweight mothers was precise and consistent, whereas for obese mothers, it was imprecise and inconsistent (Table 1).

Jiang et al. [33] systematically reviewed of maternal infection during pregnancy and risk of ASD. Their most important findings include a small but significant increase in risk of ASD after maternal bacterial (18%) and genitourinary infection (9%). The risk was precise but inconsistent. They also found a small increase in ASD after maternal flu that was precise, inconsistent, and marginally significant.

A meta-analysis of ten studies by Chen et al. [34] found that maternal autoimmune disease is associated with a small but significant, precise, and consistent increase in risk of ASD in the offspring. Similarly, a meta-analysis of 11 studies by Wu et al. [35] showed that family history of autoimmune illness increased the risk of ASD. The effect size was small and inconsistent but precise. In subgroup analysis, family history of autoimmune thyroid disease, diabetes, psoriasis, and rheumatoid arthritis were associated with an average 49–64% increase in risk of ASD in the offspring (Table 1).

Conti et al. [36] conducted a systematic review of the association between assisted reproductive technology (ART) and risk of ASD. Three out of seven studies suggested an association between ART and ASD, but these studies were of low quality. On the other hand, high-quality studies showed no association between ART and risk of ASD (Table 2).

### Medication use during pregnancy

Gentile [37] systematically reviewed the evidence for the association between maternal valproate use (a medication primarily used for epilepsy and bipolar disorder) and ASD in the offspring. Studies with both prospective and retrospective design provided strong evidence for the association of maternal valproate use and ASD as well as several other neurodevelopmental outcomes. Valproate was associated with poorer neurodevelopment than other antiepileptic drugs. Furthermore, the association seemed to be dose-related and robust to adjustment for several confounders including seizure attacks during pregnancy and maternal intelligence quotient (Table 2).

The association between maternal antidepressant use during pregnancy and ASD is more controversial. Kobayashi et al. [38] quantitatively reviewed five case-control and three cohort studies of maternal selective serotonin reuptake inhibitor (SSRI) use in pregnancy and the risk of ASD in the offspring. They found a 50% increase in risk of ASD of mothers who took SSRIs during pregnancy; the estimate was imprecise but consistent. However, when the authors conducted a sensitivity analysis comparing SSRI-exposed group to SSRI-non-exposed group in mother with psychiatric conditions, they found no significant increase in risk of ASD in the offspring. Based on this evidence, the authors concluded that the relation between SSRI and ASD finding is largely due to confounding by indication (Tables 1 and 2).

### Maternal smoking

A meta-analysis of 15 studies by Rosen et al. [39] found precise (but inconsistent) evidence that there was no association between maternal smoking and risk of ASD in the offspring. The lack of association was unaffected by adjustment for socioeconomic status and parental psychiatric history and was consistent in assessments carried out during pregnancy and at birth. For postnatal assessment of prenatal smoking, there was a slight and imprecise increase in risk, which might reflect a recall bias (Table 1).

### Nutritional factors

Several studies have tried to establish an association between nutritional elements such as folic acid or vitamin D and risk for ASD. However, many of those studies are fundamentally limited by the fact that they have assessed the deficiency and/or the efficacy of supplementing these nutrients after developing ASD. Therefore, one should be very cautious about causal interpretation of the findings in those studies.

A meta-analysis by Sharp et al. [40] showed significantly lower protein and calcium intake in children with ASD. However, the estimates were imprecise, inconsistent, and indirect. Another meta-analysis of 12 studies by Babaknejad et al. [41] showed significantly lower zinc levels in children with ASD. Wang et al. [42] performed a meta-analysis of 11 studies of the association between vitamin D and ASD. They found significantly lower levels of serum 25-hydroxy vitamin D in subjects with ASD than those in controls (Table 2). The pooled effect size was large, but inconsistent and imprecise. Of note, the number of studies that investigated vitamin D levels in maternal blood during pregnancy was too small to allow for a meta-analysis.

Systematic reviews of the association between omega-3 fatty acids and ASD have mainly focused on interventional studies. In a Cochrane review, only two small randomized trials met the eligibility criteria [43]. The authors found no evidence of a beneficial effect of omega-3 in patients with ASD (Table 2).

Castro et al. [44] systematically reviewed the evidence for folic acid involvement in risk of ASD. The authors found some evidence for the association between folate deficiency and ASD and ASD-like traits. However, the findings were inconsistent and were limited by the self-report in the majority of studies (Table 2). Another systematic review suggested that folate deficiency might interact with certain polymorphism in the methylene tetrahydrofolate reductase (MTHFR) gene to increase risk of ASD [45].

There are reports of other dietary interventions such as camel milk and casein and gluten-free diet in ASD, but studies of such interventions have generally been short term and of low quality.

### Exposure to toxins

Studies of toxic exposure have been largely limited by indirect and cross-sectional methods of exposure measurement. Quantitative systematic reviews have been performed on air pollution, thimerosal (ethylmercury), inorganic mercury, and hair levels of heavy metals. In a meta-analysis, Lam and colleagues [46] found a small but significant and precise association between prenatal exposure to particulate matters and risk of ASD. However, the risk was inconsistent across studies and the exposure was measured via indirect methods (Tables 1 and 2). Of note, the risk was substantially larger for particulate matter <2.5 μm compared to those of <10 μm.

In a meta-analysis of nine studies, Yoshimasu et al. [47] found precise and consistent evidence for lack of association between childhood thimerosal exposure and ASD. The lack of association was consistent in pooled analysis of the adjusted estimates, studies of anti-RhD antibody treatment, and studies of direct thimerosal exposure. In a meta-analysis of three case-control studies, the same authors reported a 60% increase in risk of ASD following higher level of inorganic mercury exposure. Based on their findings, the authors suggested that early life exposure to mercury by vaccination did not increase the risk of ASD, whereas exposure to inorganic mercury in the environment might be associated with an increased risk of ASD (Table 1).

De Palma and colleagues [48] performed a meta-analysis of studies that compared hair concentration of heavy metals between patients with ASD and controls. Their meta-analyses found little evidence for an association between hair metal concentration of mercury, copper, cadmium, selenium, and chromium. They did find significantly higher levels of lead in the hair of patients with ASD than those of controls. However, the estimates for lead were imprecise and inconsistent across studies and were disproportionately affected by an outlier study (Table 1).

Rossignol et al. [49] carried out the most comprehensive systematic review of environmental toxins in ASD. The authors evaluated two groups of studies based on direct or indirect assessment of exposure. Studies with indirect assessment of exposure were generally of ecological or retrospective nature and overall provide lower grade of evidence as compared with studies of more direct exposure. Most of studies of indirect exposure have shown an association between one or more environmental toxin exposures and ASD, even though the results have been inconsistent. The most consistent finding among this group of studies was for an association between environmental mercury exposure and ASD in seven of eight ecological studies (Table 2).

Rossignol and colleagues [49] also made an exhaustive effort of synthesizing the evidence on the relationship between direct biomarkers of toxic exposure and ASD. Systematic review of five metals (mercury, lead, cadmium, aluminum, and arsenic) contained eight or more case-control studies. Studies measured metal concentrations in blood, hair, tooth, urine, or brain. For all metals but arsenic, more than half of studies showed no elevation in any of the measurement in patients with ASD compared with control subjects. The authors further identified seven studies that reported an association between heavy metal concentration (mostly mercury and lead) and severity of ASD. Furthermore, 12 studies described improvement in symptoms of ASD following chelation therapy. However, none of these studies used a placebo arm and are therefore difficult to interpret. Importantly, a recent Cochrane systematic review found no evidence for the beneficial effect of chelation therapy on ASD [50] (Table 2).

Studies of the association between endocrine-disrupting chemicals and ASD are scarce. Rossignol et al. [49] reviewed two case-control studies of polychlorinated biphenyls (PCB) and found no association between PCB and risk of ASD, whereas Jeddi et al. [51] found that three out of five studies of phthalate exposure showed a significant association between phthalate exposure and risk of ASD (Table 2).

### Vaccination

Taylor et al. [52] performed a meta-analysis of studies that investigated the association between childhood vaccines and ASD. The authors found no evidence for a higher risk of ASD in subjects who are vaccinated. Their results were precise, consistent (except for case-control studies), and robust to study design or subtypes of ASD. Indeed, their findings were suggestive of a protective effect of vaccines on risk of childhood autism in case-control studies. Given the concerns over the association between ASD and the mumps, measles, and rubella (MMR) vaccine, the authors conducted a separate meta-analysis of three studies of MMR vaccine and ASD and found a non-significant decrease in risk of ASD following MMR vaccination (Table 1).

### Maternal immigration

In a meta-analysis of five studies, Gardener et al. [24] found imprecise and inconsistent association between maternal immigration and ASD. Restricting the studies to Nordic countries, they found a significant small effect size for the association between immigration and ASD. In a systematic review of the association between maternal immigration and risk of ASD, Crafa and Varfa [53] found ten eligible studies. The authors observed significantly higher risk of ASD in the immigrants in three studies, whereas five studies showed no difference between immigrant and non-immigrant mothers, and two studies showed significantly lower risk of ASD in immigrants than that in non-immigrants. After controlling for the effects of different sample sizes and ethnic backgrounds, the results showed higher rates of ASD in children of immigrant mothers (2.69%) compared with those in children of non-migrant mothers (0.91%). Six out of ten studies found that giving birth postmaternal migration significantly increased the risk of ASD, with the exception of children born to Hispanic migrant mothers who had lower rates of ASD (Tables 1 and 2).

### Possible mechanisms behind the association between environmental risk factors and ASD

A fundamental question about the association between environmental risk factors and ASD is whether the association represents an underlying causality or not. Although evidence in this area is still speculative, here, we briefly review the possible mechanisms of involvement of environmental factors in ASD. It should be emphasized that the mechanisms reviewed here are by no means exhaustive. Furthermore, each environmental factor—if causal—might involve multiple mechanisms and at different levels of etiological pathways to ASD.

### Non-causal associations

A plausible explanation for many of the observed environmental effects of ASD might be that of confounding. For example, the association between several obstetric complications and ASD might be partially confounded by parity, because parity is associated both with the exposure (i.e., birth complications) and the outcome (i.e., ASD) [54]. Moreover, a substantial proportion of what might be interpreted as causal effect of environment on ASD risk might result from a gene-environment correlation. For example, many genetic conditions that are associated with ASD might also be associated with birth complications. These epiphenomena are observed with higher frequency in children with ASD but are not necessarily causal [55]. Similarly, the association between maternal SSRI use and ASD might be due to confounding by indication. Given that ASD has a high comorbidity rate with depression, such associations might reflect a shared risk mechanism rather than causality [56]. Confounding by gene-environment correlation might also account for the association between other risk factors such as maternal immigration and ASD. It has been proposed that fathers with autistic traits are more likely to marry immigrant women [57].

### Genetic and epigenetic-related effects

In addition to gene-environment correlation, environmental factors could interact with genetic components on various levels. It has been suggested that some environmental factors such as certain toxins and vitamin D deficiency increase the risk of gene mutation that in turn can lead to an increased risk of ASD [58]. Recent evidence shows that a specific polychlorinated biphenyl congener, PCB-95, might modify the number of copy number variations leading to deletion or duplications of 15q11-q13, a genetic cause of ASD [59]. Maternal obesity modifies the expression of several important genes (such as apolipoprotein D) that are critical to neurodevelopment in utero [60].

Epigenetic mechanisms are biochemical modifications of DNA or histones that affect gene expression without changing the DNA sequence. Epigenetic mechanisms are thought to be critical in the normal development of the nervous system [61]. Some environmental risk factors of ASD might affect neurodevelopment through epigenetic mechanisms. For example, valproate, a strong risk factor for ASD, inhibits histone deacetylase and interferes with folic acid metabolism [62, 63]. Both mechanisms result in significant alterations in epigenetic modifications. Folate deficiency in a background of methylene tetrahydrofolate reductase (MTHFR) gene polymorphism might impair methyl donation and subsequently lead to impaired epigenetic regulation [64]. Assisted reproductive technology and maternal stress (due to immigration) have also been linked to epigenetic alterations [65, 66], although the importance of such association with respect to ASD is unknown.

Genetic mechanisms also serve to make the individuals susceptible to the effect of certain environmental risk factors. For example, mutation of Mecp2 gene (the cause of Rett syndrome) and a regulator of the epigenome in neurons causes social deficit in mice with prenatal exposure to the organic pollutant polybrominated diphenyl ethers (PBDE) [67]. Paraoxonase is an enzyme that metabolizes organophosphate. In a systematic review, Rossignol et al. [49] reported four studies with decreased PON1 activity and three out of five studies showing a significant association between PON polymorphism and ASD. Similarly, they observed an association between polymorphism of glutathione-S transferase (responsible for detoxification of xenobiotics and heavy metals) and ASD in three of four studies [49].

### Inflammation and oxidative stress

ASD is associated with altered immune status, increased oxidative stress, and an active neuroinflammatory process characterized by microglial activation in various parts of the brain [68–71]. A meta-analysis has indicated that concentrations of several pro-inflammatory cytokines such as interleukin-6, interluekin-1, and interferon gamma are increased in patients with ASD compared to those in healthy controls [70]. It is possible that the association of maternal autoimmune disease with risk of ASD is partly mediated through the effect of maternal inflammatory mediators and autoantibodies on fetal neurodevelopment [72, 73].

Another meta-analysis has demonstrated an association between ASD and altered metabolism of glutathione, an antioxidant [68]. Some environmental factors such as lead, mercury, persistent organic pollutants, or perinatal complications might cause a pro-inflammatory state and oxidative damage in the brain and subsequently lead to alterations in neural growth and development [69, 74–76]. Moreover, chemicals like brominated flame retardants might result in mitochondrial toxicity through a variety of mechanisms (including oxidative stress) leading to impaired energy balance in the brain [77]. Mitochondrial dysfunction has been documented in patients with ASD [78].

Deth et al. [79] has made a case for a redox/methylation hypothesis of ASD. According to this hypothesis, in a genetically sensitive individual, environmental toxins (particularly toxic metals) cause significant oxidative stress. This subsequently leads to impaired methylation and alters the capacity for synchronizing neural networks through impaired dopamine D4 receptor function. Impaired methylation also affects epigenetic mechanisms, leading to abnormal gene expression. Both mechanisms (impaired synchronization of neural networks and epigenetic alterations) are closely linked to ASD [61, 62, 80–82].

### Hypoxic-ischemic damage

Evidence shows that perinatal hypoxia and hypercarbia are associated with various neurodevelopmental outcomes including seizure, cerebral palsy, and intellectual disability [83, 84]. As we mentioned earlier, evidence suggests a role for birth asphyxia-hypoxia in risk for ASD. Lack of oxygen and acidosis associated with hypercarbia alter cell energy metabolism and subsequently lead to cell dysfunction and death [83]. Immaturity of autoregulatory mechanisms and white matter susceptibility in the neonate are important contributory factors in hypoxic-ischemic brain damage [85]. Moreover, hypoxic/ischemic insult induces inflammation, oxidative damage, and excitotoxicity all of which can lead to exacerbation of neuronal damage and death [85, 86]. Regions that are involved in cognitive function such as hippocampus and cortex are commonly injured following neonatal hypoxia [87]. A recent study has provided insight into the possible mechanism of hypoxic damage with regard to ASD [88]. The authors examined the role of fragile X mental retardation protein (FMRP) and mammalian target of rapamycin (mTOR) signaling pathway in the pathogenesis of hypoxic-ischemic encephalopathy. Hereditary impairment of FMRP is seen in cases of fragile X syndrome that is a well-recognized cause of ASD. Importantly, the authors observed an overexpression of FMRP between 36 and 39 weeks of pregnancy in the normal brain, which suggested an important physiological role for FMRP in synaptic plasticity during this period. Through comparing brain tissues of newborns with hypoxic-ischemic encephalopathy (HIE) with healthy controls, they found significantly lower FMRP expression in the brain of HIE than healthy controls. Based on these findings, the authors suggested that FMRP disruption might be one of the mechanisms through which hypoxic-ischemic damage is related to ASD.

### Endocrine disruption

ASD affects gender differentially. Not only the prevalence of ASD seems to be higher in boys but also the psychopathological, biochemical, and genetic aspects of ASD also appear to be different between males and females [89, 90]. This gender difference has been attributed to a variety of factors, including diagnostic bias in favor of males, extreme male brain (EMB) theory, and female protective effect (FPE) [90, 91]. Out of all explanations, EMB might be more related to the scope of our review. EMB theory of ASD builds on an empathizing-systematizing theory of psychological sex differences and suggests that ASD might reflect an extreme male pattern (i.e., more systematizing, less empathizing) [92]. It has been suggested that the differential effect of testosterone on sexually dimorphic brain regions might be a key mechanism to push the brain beyond that of a typical male and toward an EMB. Therefore, factors that alter hormonal balance (and particularly fetal testosterone) might contribute to risk of ASD. An important class of environmental factors that is capable of altering steroid balance is endocrine-disrupting chemicals. For example, brominated flame retardants are both associated with an increased level of free testosterone and an increased risk of ASD [93, 94]. Recent studies have shown that chemicals (such as some PCB congeners) that are associated with lower testosterone levels are also associated with lower risk of autistic behaviors [93, 95].

Another link between endocrine disruptors and ASD is alteration in thyroid function. Several studies have shown evidence of prenatal maternal thyroid dysfunction and ASD in the offspring [96, 97]. Interestingly, many endocrine-disrupting chemicals that disrupt function of thyroid hormone have also been hypothesized to increase the risk of ASD [98–102].

### Neurotransmitter alterations and abnormalities in signaling pathways

Abnormalities in glutamate, serotonin, and gamma-aminobutyric acid (GABA) have been linked to ASD [103]. Although alterations in neurotransmitter pathways can be the end result of many mechanisms we discussed above, some environmental factors interact directly with neurotransmitter pathways. For example, lead disrupts the activity of N-methyl-D-aspartate (NMDA) receptors on both pre- and postsynaptic levels [104]. Many environmental pollutants have been associated with altered glutamate levels in umbilical cord blood [105]. Similarly, it has been shown that brominated flame retardants modulate GABA in the developing nervous system [106].

Some environmental risk factors interact with intracellular signaling pathways and might pave the way to impaired neurodevelopment. Exposure to PCB and PBDE seems to alter calcium-related signaling pathway, leading to alterations in dendritic growth and subsequent abnormalities in neuronal connectivity, a key feature of ASD [106, 107]. Prostaglandin E2 (PGE2) is an important regulatory element in calcium homeostasis and synaptic plasticity in the developing brain [108, 109]. Evidence suggests disruption of PGE2 as a possible mechanism for the effect of organic chemicals, inflammation, and infection on risk of ASD [109].

### Limitations of current research

Compared to genetic studies of ASD, studies of environmental risk factors are in their infancy. Many previous studies of environmental risk factors have been limited by small sample size, retrospective or cross-sectional design, indirect measurement of exposure, and inability to ascertain exact timing of exposure with relation to a critical neurodevelopmental period. Moreover, for various reasons, most previous studies have not investigated important factors that might explain the heterogeneity of ASD such as differences in risk between males and females, differences between subtypes, and relation of symptom severity to risk factors. Importantly, the definition of ASD is very broad and encompasses multiple subtypes of the disorder, mirroring the etiological heterogeneity of the condition.

As results from large prospective cohorts or birth registries are starting to emerge, more valuable data on environmental risk factors become available. Still, heterogeneity of ASD, indirect measures, lack of a dimensional approach, and diagnostic difficulties make it challenging to draw substantial inferences from these studies. As Mandy and Lai in their state in their recent review, to understand the ASD, we must engage with its complexity and take into the account the substantial heterogeneity of the factors that affect its onset as well as its course [110]. Studies of environmental risk factors of ASD can tackle this complexity by framing ASD as a developmental psychopathology or a maladaptive response of the individual to its environment. This maladaptive response is shaped by interplay between a multitude of risk and protective factors at various levels and evolves as the result of a dynamic interaction between a person and their environment across the lifespan [110].

Furthermore, it is difficult to establish causality from observational studies due to possibility of genetic and/or environmental confounding. What is interpreted as an environmental effect might indeed be an epiphenomenon due to gene-environment correlation. Therefore, focus should be on designing studies that strengthen causal inferences of environmental risk by ruling out alternative explanation for the association. Studies that use genetically informed approach (e.g., family-based studies and in vitro fertilization designs) offer a new way to address the challenge of gene-environment correlation [111]. Moreover, gene-environment interaction and epigenetics of ASD are two areas that are clearly understudied but, as recent evidence shows, could potentially provide a substantial insight into the etiology of ASD.

## Conclusions

In the present paper, we reviewed systematic reviews and meta-analyses of environmental risk factors for ASD. Current evidence suggests that several environmental factors including vaccination, maternal smoking, thimerosal exposure, and most likely ART are unrelated to risk of ASD. Birth complications that are associated with trauma or ischemia and hypoxia have shown strong links to ASD, whereas other pregnancy-related factors such as maternal obesity, maternal diabetes, and C/S have shown a weak association with risk of ASD. Furthermore, factors such as maternal use of SSRI or C/S might be indicative of confounding by indication. The reviews on nutritional elements have yielded limited useful and/or inconclusive information about the beneficial effects of folic acid and omega-3, while vitamin D deficiency seems to be common in children with ASD. The studies on toxic elements have been largely limited by their design, but there is enough evidence for the association between some heavy metals (most important inorganic mercury and lead) and ASD that warrants further investigation. Reviews on the psychosocial risk factors for ASD are scarce, with maternal immigration being the only factor that has shown some association with ASD in systematic reviews. Biological underpinning of environmental risk factors of ASD are debated but might include non-causative association, gene-related effect, oxidative stress, inflammation, hypoxia/ischemia, endocrine disruption, neurotransmitter alterations, and interference with signaling pathways. Future studies of ASD risk factors would benefit from a developmental psychopathology approach, prospective design, precise exposure measurement, reliable timing of exposure in relation to critical developmental periods and should take into account the dynamic interplay between gene and environment by using genetically informed designs.

## Additional file

Additional file 1: Figure S1. PRISMA flow diagram and Table S1 List of excluded studies and the reason for exclusion. (DOCX 78 kb)

## Abbreviations

ART: Assisted reproductive technology
ASD: Autism spectrum disorder
C/S: Cesarean section
DZ: Dizygotic
EMB: Extreme male brain
FMRP: Fragile X mental retardation protein
FPE: Female protective effect
GABA: Gamma-aminobutyric acid
HIE: Hypoxic-ischemic encephalopathy
MMR: Mumps, measles, and rubella
MTHFR: Methylene tetrahydrofolate reductase
mTOR: Mammalian target of rapamycin
MZ: Monozygotic
NMDA: N-Methyl-D-aspartate
PBDE: Polybrominated diphenyl ethers
PCB: Polychlorinated biphenyls
PGE2: Prostaglandin E2
SSRI: Selective serotonin reuptake inhibitor

## References

[1] Association, A.P. Diagnostic and statistical manual of mental disorders (DSM-5®). 2013: American Psychiatric Pub. 10.1590/s2317-17822013000200017 24413388

[2] Baxter AJ (2015). The epidemiology and global burden of autism spectrum disorders. Psychol Med.

[3] Wingate M (2014). Prevalence of autism spectrum disorder among children aged 8 years-autism and developmental disabilities monitoring network, 11 sites, United States, 2010. MMWR Surveill Summ.

[4] Ronald A, Hoekstra RA (2011). Autism spectrum disorders and autistic traits: a decade of new twin studies. Am J Med Genet B Neuropsychiatr Genet.

[5] MacGregor AJ (2000). Twins. Novel uses to study complex traits and genetic diseases. Trends Genet.

[6] Bailey A (1995). Autism as a strongly genetic disorder: evidence from a British twin study. Psychol Med.

[7] Constantino JN, Todd RD (2000). Genetic structure of reciprocal social behavior. Am J Psychiatry.

[8] Gaugler T (2014). Most genetic risk for autism resides with common variation. Nat Genet.

[9] Hallmayer J (2011). Genetic heritability and shared environmental factors among twin pairs with autism. Arch Gen Psychiatry.

[10] Edelson LR, Saudino KJ (2009). Genetic and environmental influences on autistic-like behaviors in 2-year-old twins. Behav Genet.

[11] Hoekstra RA (2007). Heritability of autistic traits in the general population. Arch Pediatr Adolesc Med.

[12] Stilp RL (2010). Genetic variance for autism screening items in an unselected sample of toddler-age twins. J Am Acad Child Adolesc Psychiatry.

[13] Deng W (2015). The relationship among genetic heritability, environmental effects, and autism spectrum disorders: 37 pairs of ascertained twin study. J Child Neurol.

[14] Rosenberg RE (2009). Characteristics and concordance of autism spectrum disorders among 277 twin pairs. Arch Pediatr Adolesc Med.

[15] Lichtenstein P (2010). The genetics of autism spectrum disorders and related neuropsychiatric disorders in childhood. Am J Psychiatry.

[16] Kim YS, Leventhal BL (2015). Genetic epidemiology and insights into interactive genetic and environmental effects in autism spectrum disorders. Biol Psychiatry.

[17] Landrigan PJ (2010). What causes autism? Exploring the environmental contribution. Curr Opin Pediatr.

[18] Stromland K (1994). Autism in thalidomide embryopathy: a population study. Dev Med Child Neurol.

[19] Arndt TL, Stodgell CJ, Rodier PM (2005). The teratology of autism. Int J Dev Neurosci.

[20] Bandim JM (2003). Autism and Mobius sequence: an exploratory study of children in northeastern Brazil. Arq Neuropsiquiatr.

[21] Matheson SL (2011). A systematic meta-review grading the evidence for non-genetic risk factors and putative antecedents of schizophrenia. Schizophr Res.

[22] Guyatt GH (2008). GRADE: an emerging consensus on rating quality of evidence and strength of recommendations. BMJ.

[23] Wu S (2017). Advanced parental age and autism risk in children: a systematic review and meta-analysis. Acta Psychiatr Scand.

[24] Gardener H, Spiegelman D, Buka SL (2009). Prenatal risk factors for autism: comprehensive meta-analysis. Br J Psychiatry.

[25] Gardener H, Spiegelman D, Buka SL (2011). Perinatal and neonatal risk factors for autism: a comprehensive meta-analysis. Pediatrics.

[26] Kancherla V, Dennis LK (2006). A meta-analysis of prenatal, perinatal, and neonatal risks for autism. Am J Epidemiol.

[27] Kolevzon A, Gross R, Reichenberg A (2007). Prenatal and perinatal risk factors for autism: a review and integration of findings. Arch Pediatr Adolesc Med.

[28] Modabbernia A (2016). Impaired gas exchange at birth and risk of intellectual disability and autism: a meta-analysis. J Autism Dev Disord.

[29] Curran EA (2015). Research review: birth by caesarean section and development of autism spectrum disorder and attention-deficit/hyperactivity disorder: a systematic review and meta-analysis. J Child Psychol Psychiatry.

[30] Conde-Agudelo A, Rosas-Bermudez A, Norton MH. Birth spacing and risk of autism and other neurodevelopmental disabilities: a systematic review. Pediatrics. 2016;137(5). 10.1542/peds.2015-3482 27244802

[31] Xu G (2014). Maternal diabetes and the risk of autism spectrum disorders in the offspring: a systematic review and meta-analysis. J Autism Dev Disord.

[32] Wang Y (2016). Maternal body mass index and risk of autism spectrum disorders in offspring: a meta-analysis. Sci Rep.

[33] Jiang HY (2016). Maternal infection during pregnancy and risk of autism spectrum disorders: a systematic review and meta-analysis. Brain Behav Immun.

[34] Chen SW (2016). Maternal autoimmune diseases and the risk of autism spectrum disorders in offspring: a systematic review and meta-analysis. Behav Brain Res.

[35] Wu S (2015). Family history of autoimmune diseases is associated with an increased risk of autism in children: a systematic review and meta-analysis. Neurosci Biobehav Rev.

[36] Conti E (2013). Are children born after assisted reproductive technology at increased risk of autism spectrum disorders? A systematic review. Hum Reprod.

[37] Gentile S (2014). Risks of neurobehavioral teratogenicity associated with prenatal exposure to valproate monotherapy: a systematic review with regulatory repercussions. Cns Spectrums.

[38] Kobayashi T (2016). Autism spectrum disorder and prenatal exposure to selective serotonin reuptake inhibitors: a systematic review and meta-analysis. Reprod Toxicol.

[39] Rosen BN (2015). Maternal smoking and autism spectrum disorder: a meta-analysis. J Autism Dev Disord.

[40] Sharp WG (2013). Feeding problems and nutrient intake in children with autism spectrum disorders: a meta-analysis and comprehensive review of the literature. J Autism Dev Disord.

[41] Babaknejad N (2016). The relationship between zinc levels and autism: a systematic review and meta-analysis. Iran J Child Neurol.

[42] Wang T (2016). Serum concentration of 25-hydroxyvitamin D in autism spectrum disorder: a systematic review and meta-analysis. Eur Child Adolesc Psychiatry.

[43] James S, Montgomery P, Williams K (2011). Omega-3 fatty acids supplementation for autism spectrum disorders (ASD). Cochrane Database Syst Rev.

[44] Castro K (2016). Folic acid and autism: what do we know?. Nutr Neurosci.

[45] Pu D, Shen Y, Wu J (2013). Association between MTHFR gene polymorphisms and the risk of autism spectrum disorders: a meta-analysis. Autism Res.

[46] Lam J (2016). A systematic review and meta-analysis of multiple airborne pollutants and autism spectrum disorder. PLoS One.

[47] Yoshimasu K (2014). A meta-analysis of the evidence on the impact of prenatal and early infancy exposures to mercury on autism and attention deficit/hyperactivity disorder in the childhood. Neurotoxicology.

[48] De Palma G (2012). Lack of correlation between metallic elements analyzed in hair by ICP-MS and autism. J Autism Dev Disord.

[49] Rossignol DA, Genuis SJ, Frye RE (2014). Environmental toxicants and autism spectrum disorders: a systematic review. Transl Psychiatry.

[50] James S (2015). Chelation for autism spectrum disorder (ASD). Cochrane Database Syst Rev.

[51] Jeddi MZ (2016). The role of phthalate esters in autism development: a systematic review. Environ Res.

[52] Taylor LE, Swerdfeger AL, Eslick GD (2014). Vaccines are not associated with autism: an evidence-based meta-analysis of case–control and cohort studies. Vaccine.

[53] Crafa D, Warfa N (2015). Maternal migration and autism risk: systematic analysis. Int Rev Psychiatry.

[54] Bai J (2002). Parity and pregnancy outcomes. Am J Obstet Gynecol.

[55] Bolton PF (1997). Obstetric complications in autism: consequences or causes of the condition?. J Am Acad Child Adolesc Psychiatry.

[56] Ghaziuddin M, Ghaziuddin N, Greden J (2002). Depression in persons with autism: implications for research and clinical care. J Autism Dev Disord.

[57] Gillberg C, Schaumann H, Gillberg IC (1995). Autism in immigrants: children born in Sweden to mothers born in Uganda. J Intellect Disabil Res.

[58] Kinney DK (2010). Environmental risk factors for autism: do they help cause de novo genetic mutations that contribute to the disorder?. Med Hypotheses.

[59] Mitchell MM (2012). Levels of select PCB and PBDE congeners in human postmortem brain reveal possible environmental involvement in 15q11-q13 duplication autism spectrum disorder. Environ Mol Mutagen.

[60] Edlow AG (2014). Maternal obesity affects fetal neurodevelopmental and metabolic gene expression: a pilot study. PLoS One.

[61] Rangasamy S, D’Mello SR, Narayanan V (2013). Epigenetics, autism spectrum, and neurodevelopmental disorders. Neurotherapeutics.

[62] Grafodatskaya D (2010). Autism spectrum disorders and epigenetics. J Am Acad Child Adolesc Psychiatry.

[63] Balmer NV (2012). Epigenetic changes and disturbed neural development in a human embryonic stem cell-based model relating to the fetal valproate syndrome. Hum Mol Genet.

[64] Aarabi M (2015). High-dose folic acid supplementation alters the human sperm methylome and is influenced by the MTHFR C677T polymorphism. Hum Mol Genet.

[65] van Montfoort AP (2012). Assisted reproduction treatment and epigenetic inheritance. Hum Reprod Update.

[66] Franklin TB (2010). Epigenetic transmission of the impact of early stress across generations. Biol Psychiatry.

[67] Woods R (2012). Long-lived epigenetic interactions between perinatal PBDE exposure and Mecp2308 mutation. Hum Mol Genet.

[68] Frustaci A (2012). Oxidative stress-related biomarkers in autism: systematic review and meta-analyses. Free Radic Biol Med.

[69] Goines PE, Ashwood P (2013). Cytokine dysregulation in autism spectrum disorders (ASD): possible role of the environment. Neurotoxicol Teratol.

[70] Masi A (2015). Cytokine aberrations in autism spectrum disorder: a systematic review and meta-analysis. Mol Psychiatry.

[71] Vargas DL (2005). Neuroglial activation and neuroinflammation in the brain of patients with autism. Ann Neurol.

[72] Dalton P (2003). Maternal neuronal antibodies associated with autism and a language disorder. Ann Neurol.

[73] Croen LA (2008). Maternal mid-pregnancy autoantibodies to fetal brain protein: the early markers for autism study. Biol Psychiatry.

[74] Radaelli T (2003). Gestational diabetes induces placental genes for chronic stress and inflammatory pathways. Diabetes.

[75] Bastek JA, Gomez LM, Elovitz MA (2011). The role of inflammation and infection in preterm birth. Clin Perinatol.

[76] Valko M, Morris H, Cronin MT (2005). Metals, toxicity and oxidative stress. Curr Med Chem.

[77] Napoli E (2013). Toxicity of the flame-retardant BDE-49 on brain mitochondria and neuronal progenitor striatal cells enhanced by a PTEN-deficient background. Toxicol Sci.

[78] Rossignol DA, Frye RE (2012). Mitochondrial dysfunction in autism spectrum disorders: a systematic review and meta-analysis. Mol Psychiatry.

[79] Deth R (2008). How environmental and genetic factors combine to cause autism: a redox/methylation hypothesis. Neurotoxicology.

[80] Schanen NC (2006). Epigenetics of autism spectrum disorders. Hum Mol Genet.

[81] Kana RK (2009). Atypical frontal-posterior synchronization of Theory of Mind regions in autism during mental state attribution. Soc Neurosci.

[82] Paluszkiewicz SM (2011). Impaired inhibitory control of cortical synchronization in fragile X syndrome. J Neurophysiol.

[83] Scafidi J, Gallo V (2008). New concepts in perinatal hypoxia ischemia encephalopathy. Curr Neurol Neurosci Rep.

[84] Martinez-Biarge M (2011). Predicting motor outcome and death in term hypoxic-ischemic encephalopathy. Neurology.

[85] Armstrong-Wells J (2010). Neurocognitive outcomes following neonatal encephalopathy. NeuroRehabilitation.

[86] du Plessis AJ, Volpe JJ (2002). Perinatal brain injury in the preterm and term newborn. Curr Opin Neurol.

[87] de Haan M (2006). Brain and cognitive-behavioural development after asphyxia at term birth. Dev Sci.

[88] Lechpammer M, et al. Dysregulation of FMRP/mTOR signaling cascade in hypoxic-ischemic injury of premature human brain. J Child Neurol. 2016;31(4):426–32. 10.1177/0883073815596617 PMC4740274 26239490

[89] Van Wijngaarden-Cremers PJ (2014). Gender and age differences in the core triad of impairments in autism spectrum disorders: a systematic review and meta-analysis. J Autism Dev Disord.

[90] Lai MC (2013). Biological sex affects the neurobiology of autism. Brain.

[91] Halladay AK (2015). Sex and gender differences in autism spectrum disorder: summarizing evidence gaps and identifying emerging areas of priority. Mol Autism.

[92] Baron-Cohen S, Knickmeyer RC, Belmonte MK (2005). Sex differences in the brain: implications for explaining autism. Science.

[93] Braun JM (2014). Gestational exposure to endocrine-disrupting chemicals and reciprocal social, repetitive, and stereotypic behaviors in 4- and 5-year-old children: the HOME study. Environ Health Perspect.

[94] Johnson PI (2013). Associations between brominated flame retardants in house dust and hormone levels in men. Sci Total Environ.

[95] Nowack N (2015). Influence of low-level prenatal exposure to PCDD/Fs and PCBs on empathizing, systemizing and autistic traits: results from the Duisburg birth cohort study. PLoS One.

[96] Roman GC (2013). Association of gestational maternal hypothyroxinemia and increased autism risk. Ann Neurol.

[97] Yau VM (2015). Prenatal and neonatal thyroid stimulating hormone levels and autism spectrum disorders. J Autism Dev Disord.

[98] Kuo FC (2015). Relationship of urinary phthalate metabolites with serum thyroid hormones in pregnant women and their newborns: a prospective birth cohort in Taiwan. PLoS One.

[99] Giera S (2011). Individual polychlorinated biphenyl (PCB) congeners produce tissue- and gene-specific effects on thyroid hormone signaling during development. Endocrinology.

[100] Bloom MS (2014). Thyroid hormones are associated with exposure to persistent organic pollutants in aging residents of upper Hudson River communities. Int J Hyg Environ Health.

[101] Kim S (2015). Association between several persistent organic pollutants and thyroid hormone levels in cord blood serum and bloodspot of the newborn infants of Korea. PLoS One.

[102] Kim S (2013). Association between several persistent organic pollutants and thyroid hormone levels in serum among the pregnant women of Korea. Environ Int.

[103] McDougle CJ (2005). Neurochemistry in the pathophysiology of autism. J Clin Psychiatry.

[104] Neal AP, Guilarte TR (2010). Molecular neurobiology of lead (Pb(2+)): effects on synaptic function. Mol Neurobiol.

[105] Palou-Serra A (2014). Influence of prenatal exposure to environmental pollutants on human cord blood levels of glutamate. Neurotoxicology.

[106] Dingemans MM, van den Berg M, Westerink RH (2011). Neurotoxicity of brominated flame retardants: (in)direct effects of parent and hydroxylated polybrominated diphenyl ethers on the (developing) nervous system. Environ Health Perspect.

[107] Wayman GA (2012). PCB-95 modulates the calcium-dependent signaling pathway responsible for activity-dependent dendritic growth. Environ Health Perspect.

[108] Koch H (2010). Prostaglandin E2-induced synaptic plasticity in neocortical networks of organotypic slice cultures. J Neurosci.

[109] Wong CT, Wais J, Crawford DA (2015). Prenatal exposure to common environmental factors affects brain lipids and increases risk of developing autism spectrum disorders. Eur J Neurosci.

[110] Mandy W, Lai MC (2016). Annual Research Review: The role of the environment in the developmental psychopathology of autism spectrum condition. J Child Psychol Psychiatry.

[111] D’Onofrio BM (2013). Critical need for family-based, quasi-experimental designs in integrating genetic and social science research. Am J Public Health.

[112] Rais TB, Rais A (2014). Association between antidepressants use during pregnancy and autistic spectrum disorders: a meta-analysis. Inn Clin Neurosci.

[113] Sayehmiri F (2015). Zn/Cu levels in the field of autism disorders: a systematic review and meta-analysis. Iran J Child Neur.

[114] Flores-Pajot MC (2016). Childhood autism spectrum disorders and exposure to nitrogen dioxide, and particulate matter air pollution: a review and meta-analysis. Environ Res.

[115] Mihic T (2016). The therapeutic effects of camel milk: a systematic review of animal and human trials. J Evid Based Complementary Altern Med.

[116] Mari-Bauset S (2014). Evidence of the gluten-free and casein-free diet in autism spectrum disorders: a systematic review. J Child Neurol.

