# Supplementary material for: Environmental risk factors for autism: an evidence-based review of systematic reviews and meta-analyses
Source: Mol Autism. 2017 Mar 17;8:13. doi: 10.1186/s13229-017-0121-4 (PMC5356236; doi:10.1186/s13229-017-0121-4)
Supplement: Additional file 1: Figure S1. — PRISMA flow diagram and Table S1 List of excluded studies and the reason for exclusion. (DOCX 78 kb) [file 13229_2017_121_MOESM1_ESM.docx]

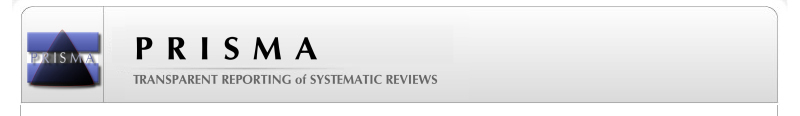
 **Figure S1: PRISMA Flow Diagram**

Studies included in synthesis
(n =32)

23 meta-analyses

9 systematic reviews

Full-text articles excluded
(n =48)

Little or no data on ASD (8)

General neurodevelopment (3)

Less comprehensive than the included study (3)

Narrative reviews (2)

No risk factors (1)

No control subjects (1)

Only in certain countries (2)

Opinion (2)

Old systematic reviews (21)

Effect on symptoms (1)

Prognostic factors (1)

Systematic review of birth cohorts (1)

Systematic review only (2)

Records excluded
(n = 584)

Biomarkers/biology (22)

Comorbidity (29)

Course/prognosis (5)

Genetic/non-environmental (82)

Incidence/prevalence (6)

Method (10)

Neuroimaging/neurophysiology (57)

Neuropsychology (51)

Not a systematic review (2)

Not ASD (47)

Reply/comment (7)

Social/functional/occupational (20)

Symptomatology/diagnosis (29)

Treatment/intervention (217)

Full-text articles assessed for eligibility
(n =80)

## Screening

## Eligibility

## Included

Records screened
(n = 664)

## Identification

Records after duplicates removed
(n = 664)

Additional records identified through other sources
(n = 1)

Records identified through Pubmed searching
(n = 664)

Table S1. List of excluded studies and the reason for exclusion

| **Study** | **Risk or protective factor(s)** | **Reason for exclusion** | **Relevant included reviews** |
| --- | --- | --- | --- |
| NPG 2006 | NA | Opinion | NA |
| Abubakar 2016 | NA | No risk factors | NA |
| Aguilar Cordero 2015 | Maternal diabetes | Systematic review only (the included study is a meta-analysis) | Xu et al 2014 |
| Alanazi et al 2013 | Nutrition | Outdated | Multiple |
| Alkandari et al. 2016 | Fetal ultrasound measurements | Little or no data on ASD | NA |
| Amin et al 2011 | Jaundice | Less comprehensive than the included study | Gardener et al 2011 |
| Bakkar et al. 2014 | Cerebrovascular function in child | Little or no data on ASD | NA |
| Bay et al 2014 | Fertility treatment | General neurodevelopment with little focus on ASD | Conti et al 2013 |
| Bent et al 2009 | Omega-3 | Outdated | James et al 2011 |
| Cimino et al 2016 | Neonicotinoid Pesticide | Little or no data on ASD | NA |
| Demicheli et al 2005 | Vaccines | Outdated | Taylor et al 2014 |
| Demicheli et al 2012 | Vaccines | Outdated | Taylor et al 2014 |
| DeVilbiss et al 2015 | Folic acid | Outdated | Castro et al 2016 |
| El Marroun et al 2014 | SSRIs | Outdated | Kobayashi et al 2016 |
| Gao et al 2016 | Folic acid | General neurodevelopment with little focus on ASD | Castro et al 2016 |
| Geier et al 2006 | Vaccine | Outdated | Taylor et al 2014 |
| Graham et al 2016 | Being refugee | Little or no data on ASD | NA |
| Grandjean et al 2014 | Toxins | Focused review (not systematic) | Multiple |
| Guxen et al 2016 | Air pollutants | Only European countries; Autistic traits | Multiple |
| Hamlyn et al 2013 | Modifiable risk factors | Narrative review | Multiple |
| Healey et al 2016 | SSRIs | Very broad definition of ASD (including neurodevelopmental disorders) | Kobayashi et al 2016 |
| Hultman et al 2011 | Advancing parental age | Outdated | Wu et al 2017 |
| Hvidtjorn et al 2009 | Assisted conception | Outdated | Conti et al 2013 |
| James et al 2015 | Chelation | Only 1 study of ASD | NA |
| Jefferson et al 2003 | Vaccine | Outdated | Taylor et al 2014 |
| Kaplan et al 2016 | SSRI | Smaller number of pooled studies than the included study | Kobayashi et al 2016 |
| Li et al . 2015 | Maternal Obesity | Outdated | Wang et al 2016 |
| Linsell et al 2016 | VLBW, Very preterm | Prognostic factors | NA |
| Maglione et al 2014 | Vaccine | Only in the US; no meta-analysis | Taylor et al 2014 |
| Main et al 2010 | Folic acid | Outdated | Castro et al 2016 |
| Man et al 2015 | SSRI | Outdated | Kobayashi et al 2016 |
| McDermott et al 2015 | Nickel and Chromium during pregnancy | Only 1 study of ASD | NA |
| McDonagh et al 2014 | SSRI | Outdated | Kobayashi et al 2016 |
| Millward et al 2004 | Gluten and casein free diet | Outdated | Mari-bauset et al 2014 |
| Millward et al 2008 | Gluten and casein free diet | Outdated | Mari-bauset et al 2014 |
| Morales-Suarez-Varela et al 2016 | Particulate matter | Systematic review only (the included study is a meta-analysis) | Multiple |
| Ng et al 2007 | Mercury | Outdated | Yoshimasu et al 2014 |
| Nye et al 2002 | B6-Mg | Only 1 study of ASD | NA |
| Rucklidge et al 2013 | Micronutrients | Outdated | Multiple |
| Sandin et al 2012 | Advancing parental age | Outdated | Wu et al 2017 |
| Tang et al 2015 | Smoking | Less comprehensive than the included study | Rosen et al 2015 |
| Thompson et al | All risk factors in the birth cohorts | Systematic review of birth cohorts | Multiple |
| Ticher et al 1996 | Circannual pattern | No control subjects | NA |
| Turville et al 2015 | Vaccine | Opinion | Taylor et al 2014 |
| Uljarevic et al 2016 | Multilingualism | Effect on symptoms | NA |
| Wang et al 2016 | Probiotic | Only 1 study | NA |
| Wilson et al 2013 | Vaccines | Outdated | Taylor et al 2014 |
| Zibaee et al 2015 | Camel Milk | Outdated | Mihic et al 2016 |
